# Brace roots in C _3_ Poaceae: where have they gone?

**DOI:** 10.17912/micropub.biology.000939

**Published:** 2023-09-20

**Authors:** Anna Siosiou, Erin E. Sparks, Ioannis T. Tsialtas

**Affiliations:** 1 Faculty of Agriculture, Lab. of Agronomy, Aristotle University of Thessaloniki, Thessaloniki, Central Macedonia, Greece; 2 Department of Plant and Soil Sciences, University of Delaware, Newark, Delaware, United States

## Abstract

Brace roots are common in large C _4_ Poaceae species, such as maize and sorghum. However, in other species, these roots were either never reported, or the existence of the trait was neglected. Here we report the presence of brace roots in a high-performing *Avena sativa* L. (oat) line.

**Figure 1.**
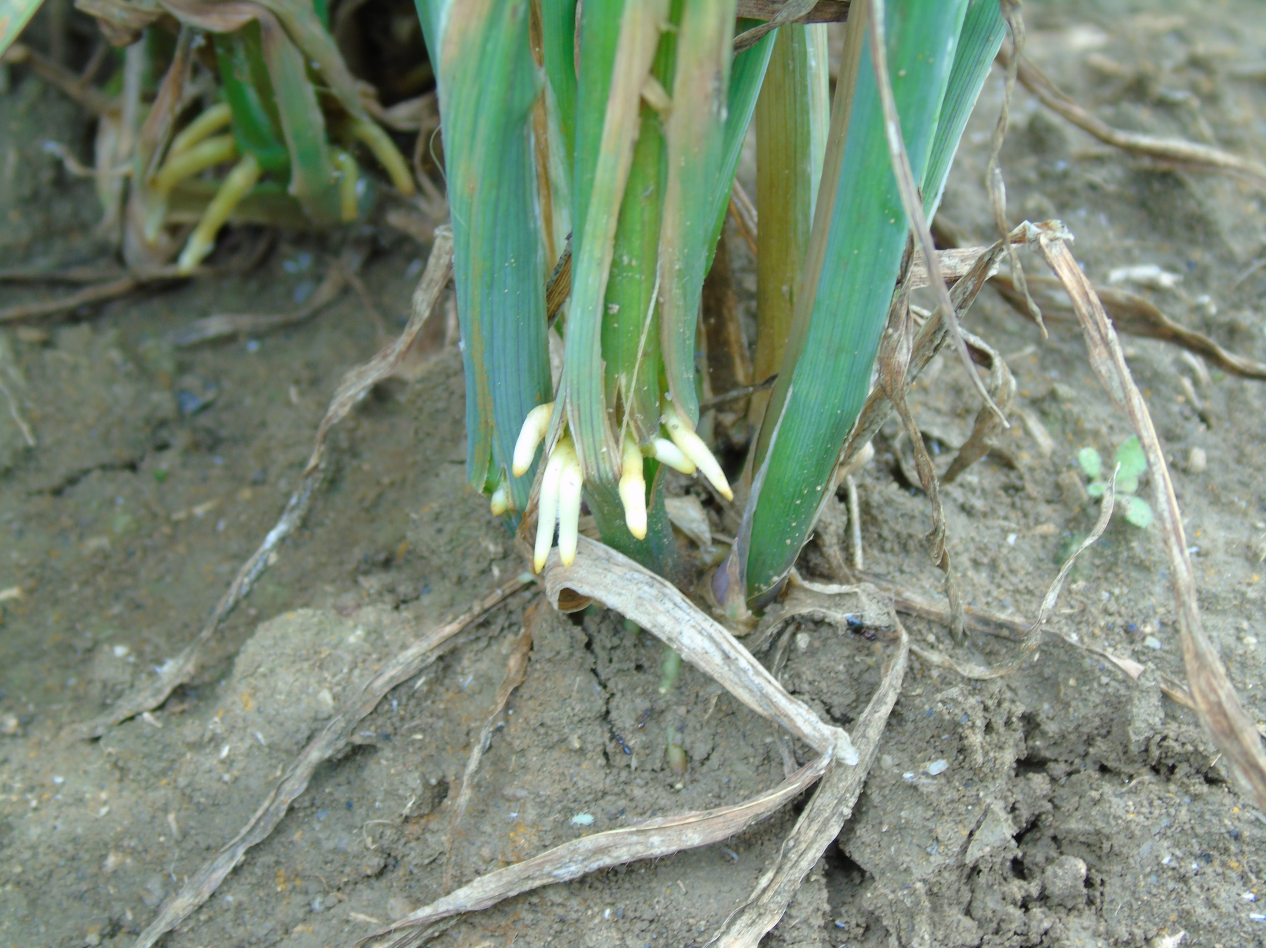
Brace roots on oat plant grown in the field.

## Description

Brace roots are aerial nodal roots that are a type of adventitious roots developed from the stem nodes aboveground (Steffens & Rasmussen 2016) . The belowground nodal roots are called crown roots. The stages and the effectors of nodal root development are still under research, and day by day the knowledge is enriched (Hostetler et al. 2021) .

The developmental mechanism that is widely supported indicates the diversification of the cells on the node. Then, primordia are formed within the stem node and stay in a latent phase until the signal to emerge. When this happens, the primordia will elongate and develop from the node. For the aboveground nodal roots, they continue to elongate until they reach the soil where they function to brace the plant (Atkinson et al. 2014; Hostetler et al. 2021) .

The plant’s main profit when developing brace roots is the mechanical reinforcement of the stems, so they can grow with low percentage in lodging (the term for mechanical failure in agricultural plants) (Hostetler et al. 2022) . Other benefits regard the better intake of nutrients and water (Lynch 2013; 2019) and putatively, it can be a defense mechanism to drought or flooding. Thus, brace roots are not only involved in water uptake, but also influence the root system health (Sauter 2013; Sharma & Carena 2016) .

The most reviewed brace roots are the ones that *Zea* *mays* L. (corn or maize) grows, with many advantages such as resistance to root lodging and better nitrogen and water uptake. Brace roots are also referenced in other C _4_ Poaceae like *Sorghum bicolor* L *.* , with similar functions as stated previously (Li et al. 2014; Hostetler et al. 2021) . The developmental mechanisms of brace roots are likely shared with the crown roots (Hostetler et al. 2021) . Thus, any species that develops crown roots should be able to develop brace roots, but there was little evidence, until now, to confirm that (Hostetler et al. 2021) .

*Avena sativa* L. (oat) is an important Poaceae crop grown for food and forage. Like other Poaceae, oats rely on nodal roots for their root function. In 1960s and 1970s, two registered varieties of oat (Nodaway and Nodaway 70) were described to develop brace roots that aid in lodging-resistance (Poehlman 1962; Poehlman 1971) , but to our knowledge brace roots have not been reported in oats in over 50 years since these reports.

In the spring 2023, in a field experiment of intercropping, an accession of *Avena sativa* L. with *Vicia sativa* L. ( *sensu lato* vetch) accessions, the oat plants consistently developed brace roots. There were brace roots growing from the stem, mostly from the first node aboveground of the outer tillers ( Figure 1 ). The oat accession was an advanced line selected for use as a forage. The plants that developed brace roots were tall (> 1.00 m), with thick stems and many tillers, but showing no lodging despite being grown on an alkaline (pH 7.87), heavy, fertile loam (organic matter >2.0% and 46.0 mg NO _3_ -N/kg). Interestingly, the same accession at the same site grew shorter (ca. 80 cm) and showed no brace roots.

We show that brace roots can also develop in oats, although the development is environmentally regulated. In particular, the intercropping with vetch may trigger plant competition, which induces the outgrowth of brace roots. Further, the high soil nitrogen may also trigger the outgrowth of brace roots. This has been reported in maize, where increased planting density reduces brace root outgrowth and high nitrogen levels increase brace root outgrowth (Pellerin 1994) . In this case, the environment triggers the outgrowth of existing brace root primordia. It remains unclear if all oat accession also make primordia that can be triggered to grow under the right conditions. We believe this is unlikely since the observation of brace roots is limited, and propose there is an additional genetic component that renders this accession competent to make primordia that then grow under the right environmental triggers.

Overall, these data support our proposition that any species that develops crown roots should be able to develop brace roots and urges us to search the extent this attribute occurs not only in oat, but also in C _3_ Poaceae. Further research into the effectors triggering their formation and the putative benefits for plants is needed. Focusing on oat, a crop that often suffers heavily from lodging, clarifying the development of brace roots and their triggers can lead to the breeding and selection of cultivars tolerant to lodging under high resource (nutrients, water) conditions and thus, to better yields. Possibly, other C _3_ Poaceae species develop brace root too, but the trait or cropping conditions negate the triggers.

## Methods

**A field experiment of intercropping oat ( *Avena sativa* L.) with *Vicia sativa* L. subspecies was established on 29 November 2022 in the farm (** 40°32´1 N, 22°59´3 E, 0 m a.s.l.) **of Aristotle University of Thessaloniki (** AUTh), Thermi, Greece. **The soil is typic xerorthent and the climate is classified as a cold semiarid climate (Köppen: BSk) and considered to be at the edge of the category of Mediterranean climates (Vlachostergios et al. 2021) . The oat accession used was an advanced line selected by the Lab. of Agronomy, Faculty of Agriculture, AUTh. Sowing was conducted by hand at a rate of 200 seeds/m ^2^ in monocultures and 50 seeds/m ^2^ in intercrops. Apart from hand-weeding, no other agronomic practice (e.g. fertilization or irrigation) was applied after seeding.**

**The formation of brace roots was observed during biomass harvests (late April to early May). In monocultures, oat was harvested at the end of heading (BBCH 59 stage, Lancashire et al. 1991), while in the intercrops, harvests took place at the full-bloom of vetch (BBCH 65 stage, Enriquez-Hidalgo et al. 2020), when oats were at late boot (BBCH 45 stage) to heading stages (BBCH 59 stage). The brace roots were found both in intercrops and in monoculture, while they were absent in the plants of the same accession in a nearby location where oats grown shorter.**

**The height of 10 main stems (ground level to flag leaf) were measured with a common ruler in the intercropping experiment and in the neighboring site where the same accession was grown but the brace roots were absent.**
